# A Genome-Wide Gene-Based Gene–Environment Interaction Study of Breast Cancer in More than 90,000 Women

**DOI:** 10.1158/2767-9764.CRC-21-0119

**Published:** 2022-04-08

**Authors:** Xiaoliang Wang, Hongjie Chen, Pooja Middha Kapoor, Yu-Ru Su, Manjeet K. Bolla, Joe Dennis, Alison M. Dunning, Michael Lush, Qin Wang, Kyriaki Michailidou, Paul D.P. Pharoah, John L. Hopper, Melissa C. Southey, Stella Koutros, Laura E. Beane Freeman, Jennifer Stone, Gad Rennert, Rana Shibli, Rachel A. Murphy, Kristan Aronson, Pascal Guénel, Thérèse Truong, Lauren R. Teras, James M. Hodge, Federico Canzian, Rudolf Kaaks, Hermann Brenner, Volker Arndt, Reiner Hoppe, Wing-Yee Lo, Sabine Behrens, Arto Mannermaa, Veli-Matti Kosma, Audrey Jung, Heiko Becher, Graham G. Giles, Christopher A. Haiman, Gertraud Maskarinec, Christopher Scott, Stacey Winham, Jacques Simard, Mark S. Goldberg, Wei Zheng, Jirong Long, Melissa A. Troester, Michael I. Love, Cheng Peng, Rulla Tamimi, Heather Eliassen, Montserrat García-Closas, Jonine Figueroa, Thomas Ahearn, Rose Yang, D. Gareth Evans, Anthony Howell, Per Hall, Kamila Czene, Alicja Wolk, Dale P. Sandler, Jack A. Taylor, Anthony J. Swerdlow, Nick Orr, James V. Lacey, Sophia Wang, Håkan Olsson, Douglas F. Easton, Roger L. Milne, Li Hsu, Peter Kraft, Jenny Chang-Claude, Sara Lindström

**Affiliations:** 1 Department of Epidemiology, School of Public Health, University of Washington, Seattle, Washington.; 2 Public Health Sciences Division, Fred Hutchinson Cancer Research Center, Seattle, Washington.; 3 Division of Cancer Epidemiology, German Cancer Research Center (DKFZ), Heidelberg, Germany.; 4 Faculty of Medicine, University of Heidelberg, Heidelberg, Germany.; 5 Department of Public Health and Primary Care, Centre for Cancer Genetic Epidemiology, University of Cambridge, Cambridge, United Kingdom.; 6 Department of Oncology, Centre for Cancer Genetic Epidemiology, University of Cambridge, Cambridge, United Kingdom.; 7 Biostatistics Unit, The Cyprus Institute of Neurology and Genetics, Nicosia, Cyprus.; 8 Cyprus School of Molecular Medicine, Nicosia, Cyprus.; 9 Centre for Epidemiology and Biostatistics, School of Population and Global Health, The University of Melbourne, Melbourne, Victoria, Australia.; 10 Precision Medicine, School of Clinical Sciences at Monash Health, Monash University, Clayton, Victoria, Australia.; 11 Cancer Epidemiology Division, Cancer Council Victoria, Melbourne, Victoria, Australia.; 12 Department of Clinical Pathology, The University of Melbourne, Victoria, Australia.; 13 Division of Cancer Epidemiology and Genetic, NCI, NIH, Bethesda, Maryland.; 14 Genetic Epidemiology Group, School of Population and Global Health, University of Western Australia, Crawley, Australia.; 15 Department of Community Medicine and Epidemiology, Carmel Medical Center, Haifa, Israel.; 16 Cancer Control Research, BC Cancer and School of Population and Public Health, University of British Columbia, Vancouver, Canada.; 17 Public Health Sciences, Queen's University, Kingston, Canada.; 18 Université Paris-Saclay, Inserm, CESP, Team Exposome and Heredity, Villejuif, France.; 19 Department of Population Science, American Cancer Society, Atlanta, Georgia.; 20 Genomic Epidemiology Group, German Cancer Research Center (DKFZ), Heidelberg, Germany.; 21 Division of Clinical Epidemiology and Aging Research, German Cancer Research Center (DKFZ), Heidelberg, Germany.; 22 Dr. Margarete Fischer-Bosch-Institute of Clinical Pharmacology, Stuttgart, Germany.; 23 University of Tübingen, Tübingen, German.; 24 Translational Cancer Research Area, University of Eastern Finland, Kuopio, Finland.; 25 Institute of Clinical Medicine, Pathology and Forensic Medicine, University of Eastern Finland, Kuopio, Finland.; 26 Biobank of Eastern Finland, Kuopio University Hospital, Kuopio, Finland.; 27 Institute for Medical Biometry and Epidemiology, University Medical Center Hamburg-Eppendorf, Hamburg, Germany.; 28 Center for Genetic Epidemiology, Department of Preventive Medicine, Keck School of Medicine, University of Southern California, Los Angeles, California.; 29 Epidemiology Program, University of Hawaii Cancer Center, Honolulu, Hawaii.; 30 Department of Health Sciences Research, Mayo Clinic, Rochester, Minnesota.; 31 Genomics Center, Centre Hospitalier Universitaire de Québec-Université Laval Research Center, Québec City, Quebec, Canada.; 32 Department of Medicine, McGill University, Montréal, Quebec, Canada; Division of Clinical Epidemiology, Royal Victoria Hospital, McGill University, Montréal, Quebec, Canada.; 33 Division of Epidemiology, Vanderbilt University Medical Center, Nashville, Tennessee.; 34 Lineberger Comprehensive Cancer Center, University of North Carolina at Chapel Hill, Chapel Hill, North Carolina.; 35 Channing Division of Network Medicine, Department of Medicine, Brigham & Women's Hospital, Boston, Massachusetts.; 36 Department of Population Health Sciences, Weill Cornell Medicine, New York, New York.; 37 Department of Epidemiology, Harvard T.H. Chan School of Public Health, Boston, Massachusetts.; 38 Usher Institute of Population Health Sciences and Informatics, University of Edinburgh Medical School, Edinburgh, United Kingdom.; 39 Division of Evolution and Genomic Medicine, School of Biological Sciences, Faculty of Biology, Medicine and Health, Manchester Academic Health Science Centre, University of Manchester, Manchester, United Kingdom.; 40 Genomic Medicine, St Mary's Hospital, Manchester Centre for Genomic Medicine, Manchester University Hospitals NHS Foundation Trust, Manchester Academic Health Science Centre, Manchester, United Kingdom.; 41 NIHR Manchester Biomedical Research Centre, Manchester Academic Health Science Centre, Manchester University NHS Foundation Trust, Manchester, United Kingdom.; 42 Division of Cancer Sciences, University of Manchester, Manchester, United Kingdom.; 43 Department of Medical Epidemiology and Biostatistics, Karolinska Institutet, Stockholm, Sweden.; 44 Institute of Environmental Medicine, Karolinska Institutet, Stockholm, Sweden.; 45 Epidemiology Branch, Division of Intramural Research, National Institute of Environmental Health Sciences, National Institute of Health, Research Triangle Park, North Carolina.; 46 Division of Genetics and Epidemiology, The Institute of Cancer Research, London, United Kingdom.; 47 Division of Breast Cancer Research, The Institute of Cancer Research, London, United K.ingdom; 48 Centre for Cancer Research and Cell Biology, Queen's University Belfast, Belfast, United Kingdom.; 49 Department of Population Sciences, Beckman Research Institute of City of Hope, Duarte, California.; 50 Departments of Oncology and Cancer Epidemiology, Clinical Sciences, Lund University, Lund, Sweden.; 51 Department of Biostatistics, School of Public Health, University of Washington, Seattle, Washington.; 52 Program in Genetic Epidemiology and Statistical Genetics, Harvard T.H. Chan School of Public Health, Boston, Massachusetts.; 53 Cancer Epidemiology Group, University Medical Centre Hamburg-Eppendorf, University Cancer Centre Hamburg (UCCH), Hamburg, Germany.; † Deceased.

## Abstract

Genome-wide association studies (GWAS) have identified more than 200 susceptibility loci for breast cancer, but these variants explain less than a fifth of the disease risk. Although gene–environment interactions have been proposed to account for some of the remaining heritability, few studies have empirically assessed this.

We obtained genotype and risk factor data from 46,060 cases and 47,929 controls of European ancestry from population-based studies within the Breast Cancer Association Consortium (BCAC). We built gene expression prediction models for 4,864 genes with a significant (*P* < 0.01) heritable component using the transcriptome and genotype data from the Genotype-Tissue Expression (GTEx) project. We leveraged predicted gene expression information to investigate the interactions between gene-centric genetic variation and 14 established risk factors in association with breast cancer risk, using a mixed-effects score test.

After adjusting for number of tests using Bonferroni correction, no interaction remained statistically significant. The strongest interaction observed was between the predicted expression of the *C13orf45* gene and age at first full-term pregnancy (P_GXE_ = 4.44 × 10^−6^).

In this transcriptome-informed genome-wide gene–environment interaction study of breast cancer, we found no strong support for the role of gene expression in modifying the associations between established risk factors and breast cancer risk.

Our study suggests a limited role of gene–environment interactions in breast cancer risk.

## Introduction

Breast cancer is the most commonly diagnosed malignancy in women. In 2020, breast cancer was estimated to be newly diagnosed in 2.3 million women, and meanwhile caused more than 680,000 deaths worldwide (1). Both genetic and environmental factors have been found to contribute to the etiology of breast cancer. Twin studies have estimated that approximately 30% of variance in breast cancer incidence can be explained by genetic variation (2, 3). Genome-wide association studies (GWAS) have identified more than 200 independent loci that are associated with breast cancer risk (4). However, these single-nucleotide polymorphisms (SNPs) only explain approximately 19% of the familial relative risk. Meanwhile, observational studies have demonstrated that several environmental and lifestyle risk factors, including age at menarche, body mass index (BMI), alcohol consumption, parity, and use of menopausal hormone therapy (MHT), also affect the risk of breast cancer (5–11). Exploring the interplay of genetic and environmental risk factors (GxE interactions) is thus crucial in understanding the development of breast cancer.

The Breast Cancer Association Consortium (BCAC) has published multiple studies which reported various interactions between individual SNPs and established risk factors. Nickels and colleagues reported potential interactions between genetic variants and several environmental and lifestyle factors, including number of full-term pregnancies, alcohol consumption, and ever being parous (12). Schoeps and colleagues reported that two SNPs on locus *21q22.12* may interact with postmenopausal BMI to significantly affect the risk of breast cancer (13). However, other previous genome-wide gene–environmental interaction studies (GWEIS) reported no statistically significant interactions between SNPs and established breast cancer risk factors (4, 14–20). Statistical power remains one of the primary issues in GWEIS, as they require much larger sample sizes for detecting interactions as compared with marginal associations of similar magnitude (4, 21).

Novel statistical methods, such as gene-based testing that incorporates functional information, can substantially reduce the burden of multiple comparisons. As most GWAS hits fall outside of the coding region of genes and are enriched in regulatory elements, it has been hypothesized that many GWAS-identified genotype–phenotype associations are driven by the regulatory function on the expression of nearby genes (22–24). Wu and colleagues conducted a transcriptome-wide association study (TWAS) of breast cancer that systematically investigated the association between predicted gene expression and disease risk, and reported 48 statistically significant genes associations (25). These results suggest that incorporating SNP-specific regulatory information on gene expression could help discovering meaningful GxE interactions.

In this study, we utilized the genotype and environmental risk factor data collected by the Breast Cancer Association Consortium (BCAC). Using breast tissue–specific transcriptome and genotype data from the Genotype-Tissue Expression (GTEx) project, we built gene expression prediction models for 4,864 genes with a significant heritable component. We then systematically assessed the interactions between these genes and 14 established risk factors in relation to the risk of breast cancer, using a mixed-effects score test called MiSTi (mixed-effects score test for interactions; ref. 26). Our study is the first to incorporate genetically determined gene expression data in the investigation of GxE interactions in breast cancer.

## Materials and Methods

### Study Sample

For this study, we obtained breast cancer cases and controls from the cohort studies and population-based case–control studies participating in BCAC. BCAC is a well-established, international collaborative consortium of 84 epidemiologic and clinical breast cancer studies, which is integrated by investigators interested in the inherited risk of breast cancer (4). Genotype data were generated using either the iCOGs or OncoArray genotyping platforms. Both SNP arrays were customized and manufactured by Illumina, and consisted of 211,155 (iCOGs) and 533,000 (OncoArray) SNPs, respectively. In total, our study included 93,989 women (73,441 genotyped by OncoArray, 20,548 genotyped by iCOGS) from 31 studies, including ABCFS (27), AHS (28), BCEES (29), BCINIS (30, 31), CBCS (32–35), CECILE (36), CPSII (37), CTS (38), EPIC (39), ESTHER (40), GENICA (41, 42), GESBC (43), KARMA (44), KBCP (45, 46), MARIE (47), MCCS (48), MEC (49), MISS (50, 51), MMHS (52), MTLGEBCS (53), NBHS (54), NCBCS (55, 56), NHS (57), NHS2 (58), PBCS (59), PLCO (60), PROCAS (61), SASBAC (62), SISTER (63, 64), SMC (65), and UKBGS (ref. 66; Supplementary Table S1). In total, our study included 46,060 breast cancer cases (35,561 genotyped by OncoArray, 10,499 genotyped by iCOGs) and 47,929 controls (37,880 genotyped by OncoArray, 10,049 genotyped by iCOGs). All the women included were of European ancestry.

Details of the genotype calling, imputation, and quality control processes have been described elsewhere (67). Genotypes were imputed for all samples using the October 2014 (version 3) release of the 1000 Genomes Project dataset as the reference panel. The imputation was conducted using a two-stage approach, using SHAPEIT2 for phasing and IMPUTEv2 for imputation. Approximately 11.8 million SNPs with minor allele frequency (MAF) > 0.5% and imputation quality score (INFO) > 0.3 were included in our analysis.

### Building the Prediction Model of Gene Expression

We used the RNA-sequencing and genotype data from 251 individuals published by the GTEx project version 7 to construct prediction models of gene expression in mammary tissue. Details of the GTEx project have been described elsewhere (68).

We built gene expression prediction models for each gene using the “FUSION” pipeline. Only the 1,217,312 SNPs included in the HapMap Phase 3 were included in building the prediction models. To estimate the genetically modulated expression of each gene, we included variants located within 500 kb on either side of the gene boundary. SNP-heritability of each gene was estimated using the REML algorithm implemented in the GCTA software (69). Gene expression models were constructed only if the SNP-heritability of gene expression was statistically significant at *P* < 0.01. Three prediction schemes, single best eQTL (Top1), LASSO regression, and elastic-net regression, were then utilized to build expression models for each heritable gene. The prediction accuracy of each derived model was then estimated using 5-fold cross-validation, and the best performing model was selected as the final model for each gene. We built gene expression prediction models for a total of 5,043 genes, of which we had breast cancer genotype data for 4,864 genes. The gene expression prediction models were then used as functional weights in the subsequent interaction analyses.

### Collection of Breast Cancer Risk Factors

All demographic and breast cancer risk factor data were self-reported via interview or questionnaire prior to or shortly after breast cancer diagnosis (for cases) or the reference date (for controls, defined as the diagnosis date of matched breast cancer case). A total of 14 risk factors were included in the present analysis: age at first full-term pregnancy (per 5-year), average lifetime alcohol consumption (per 10 g/day), age at menarche (per 2-year), premenopausal BMI (per 5 kg/m^2^), postmenopausal BMI (per 5 kg/m^2^), breastfeeding history (yes/no), duration of breastfeeding (per 12-month), height (per 5 cm), history of oral contraceptive (OC) use (yes/no), parous (yes/no), number of full-term births (1/2/3/4+), current smoking status, current use of estrogen only (E-only) MHT, and current use of estrogen plus progestogen (E+P) MHT. BMI was analyzed separately for pre- and postmenopausal women, as the association between BMI and breast cancer risk varies across life stages (70). Analyses of reproductive factors were limited to parous women only and analyses of MHT use were limited to postmenopausal women.

### Investigating Interactions between Predicted Gene Expression and Environmental Factors

We utilized a mixed-effects based analysis tool “MiSTi” (mixed-effects score test for interactions) to assess potential GxE interactions (26). MiSTi is a hierarchical model that assesses the joint interactions of a set of variants with environmental factors, by leveraging functional information across the variants. The GxE interaction is modeled by two components, one fixed and one random effects component. The fixed-effect component incorporates variant-specific functional information as weights to calculate the weighted burden of the variants, and then quantifies their interaction with the environmental factor. The random effects component involves any residual GxE interaction effect that cannot be addressed by the fixed effects. Here, the fixed effect component represents the interaction between predicted gene expression and the environmental factor, whereas the random effects component represents the residual interaction effects of any SNPs that were not accounted for in predicted gene expression. MiSTi includes a novel testing procedure, which derives two independent score statistics for the fixed effect and the random variance component separately and combines these two statistics through an adaptive weighted linear combination (aMiSTi) to assess the evidence of overall GxE interactions. The statistical power for GxE interaction analysis using MiSTi may be affected by multiple factors, including the LD structure of the gene, proportion of the variation in gene expression explained by the genetic regulatory variants, consistency of direction of effect between random and fixed effect, etc (71). Simulation analysis suggested that under type I error rate of 0.05, a sample size of 5,000 cases and 5,000 controls, for a gene harboring 100 genetic variants of which 27 were functional, MiTIi had an 81.3% of power to detect a significant GxE interaction using the aMiSTi approach when the fixed and random component had the same direction of interaction effect (26).

In each GxE interaction model, we adjusted for study, age (at diagnosis for cases; at reference date for controls), and first five principal components for population structure. For tests of current MHT use (E-only and E+P), we further adjusted for former use of the corresponding MHT (yes/no) in the model, to account for the association between former use of MHT (which attenuates with time since cessation) and breast cancer. To adjust for multiple comparisons, we considered any interactions with aMiSTi p-value < 0.05/(4,864 × 14) = 7.34 × 10^−7^ as statistically significant. Because Bonferroni correction makes the strong assumption of independent tests and results in a stringent threshold for significance, we also report GxE interactions with a *P* value corresponding to a false discovery rate (FDR)<0.2 using the Benjamini–Hochberg (BH) approach as suggestive findings.

### Data Availability Statement

The data generated in this study are available upon request from the corresponding author.

## Results

The distribution of environmental factors in the study sample is summarized in Table 1. Compared with the control sample, breast cancer cases had a relatively higher lifetime alcohol consumption (6.5 vs. 5.7 g/day), and were less likely to be parous (85.9% vs. 88.3%). For the parous women, cases were less likely to have ever breastfed (77.5% vs. 78.7%) and reported shorter duration of breastfeeding (8.3 vs. 9.0 months). Among postmenopausal women, cases were more likely than controls to be current users of E+P MHT (18.4% vs. 12.6%) but less likely to be current users of E-only MHT (15.6% vs. 16.9%). No substantial difference was found between cases and controls for other risk factors, including age at menarche, age at first full-term birth, pre- and postmenopausal BMI, adult height, number of full-term births, OC use, and smoking status. Associations between environmental factors and breast cancer risk quantified by logistic regression are shown in the Supplementary Table S2.

**TABLE 1:** Distribution of environmental variables in the study population.

Continuous variables				
	Cases		Controls	
Variable name	Sample size	Mean (SD)	Sample size	Mean (SD)
Age at menarche, y	43,138	12.91 (1.55)	45,513	12.99 (1.56)
Age at first full-term pregnancy^a^, y	35,419	24.98 (4.67)	39,038	24.68 (4.55)
Duration of breastfeeding^a^, mo	20,425	8.34 (10.96)	18,853	8,97 (11.35)
Adult BMI, Premenopausal^b^, kg/m^b^	11,420	25.57 (5.47)	11,940	25.41 (5.17)
Adult BMI, Postmenopausal^c^, kg/m^b^	31,036	26.78 (5.32)	33,213	26.39 (5.08)
Adult Height, cm	41,819	163.79 (6.45)	45,073	163.76 (6.45)
Lifetime alcohol consumption, g/day	22,653	6.53 (12.36)	21,337	5.72 (10.50)
**Categorical variables**				
	**Cases**		**Controls**	
**Variable name**	**Sample size**	**%**	**Sample size**	**%**
Parity	43,465		45,771	
Parous	37,315	85.9	40,394	88.3
Nulliparous	6,150	14.1	5,377	11.7
Number of full-term births^a^	36,906		40,188	
1	6,714	18.2	6,147	15.3
2	15,578	42.2	16,966	42.2
3	8,910	24.1	10,061	25.0
4+	5,704	15.5	7,014	17.5
Ever breastfed^a^	25,135		23,561	
Yes	19,491	77.5	18,532	78.7
No	5,644	22.5	5,029	21.3
Ever use of OCs	41,359		43,269	
Yes	23,905	57.8	25,825	59.7
No	17,454	42.2	17,444	40.3
Smoking status	39,340		41,804	
Current	5,674	14.4	5,746	13.8
Former	12,136	30.9	12,845	30.7
Never	21,530	54.7	23,213	55.5
MHT use, Estrogen + Progestogen^c^	17,128		16,904	
Current	3,159	18.4	2,139	12.6
Former	1,557	9.1	1,554	9.2
Never	12,412	72.5	13,211	78.2
MHT Use, Estrogen^c^	17,163		16,911	
Current	2,685	15.6	2,855	16.9
Former	2,221	12.9	2,124	12.6
Never	12,257	71.5	11,932	70.5

^a^Among women with at least one full-term birth only.

^b^Among premenopausal women only.

^c^Among postmenopausal women only.

The full list of GxE interaction results is reported in Supplementary Table S3.1–S3.14. Quantile–quantile plots of aMiSTi *P* values for GxE interactions are shown in Supplementary Fig. S1. We observed an inflation of interaction test statistics for current use of E-only and E+P MHT and thus, any results for MHT use should be interpreted with caution. Overall, no interactions remained statistically significant after adjusting for number of tests performed using Bonferroni correction. The strongest evidence of interaction was observed for the *C13orf45* gene on chromosome 13 and age at the first full-term pregnancy (Table 2, *P*_GXE_ = 4.44 × 10^−6^). The heritability of *C13orf45* expression was estimated to 0.21, based on 580 SNPs. However, the interaction was mainly driven by the random effects component (*P* = 1.03 × 10^−6^) rather than fixed effects component (*P* = 0.62), which indicates there may be some SNP interaction effects that are beyond the predicted gene expression. Six additional GxE interactions were identified with an FDR-corrected *P*_GXE_ < 0.2 (Table 2). These included interactions between *RP11–219D15.3* (3q23) and age at menarche (*P*_GXE_ = 1.60 × 10^−5^); *EML4* (2p21) and use of OCs (*P*_GXE_ = 2.91 × 10^−5^); history of breastfeeding and *AC114730.3* (2q37.3, *P*_GXE_ = 6.85 × 10^−5^) and *AKAP3* (12p13.32, *P*_GXE_ = 3.58 × 10^−5^) in parous women; smoking status and *PMS2P3* (7q11.23, *P*_GXE_ = 4.00 × 10^−5^), and *RP11–7I15.4* (11q14.1, *P*_GXE_ = 6.94 × 10^−5^).

**TABLE 2:** Suggestive interactions between genes and environmental risk factors, with FDR-corrected adaptive weighted *P* < 0.20.

				*P* values			
Environmental risk factors	Gene name	CHR	# of SNPs	Fixed effect	Random effect	Adaptive weighted	FDR-corrected, Adaptive weighted^a^
Age at first full-term pregnancy	*C13orf45*	13q22.2	580	6.24E-01	1.03E-06	4.44E-06	0.02
Age at menarche	*RP11–219D15.3*	3q23	424	5.51E-06	1.07E-01	1.60E-05	0.08
Use of OC	*EML4*	2p21	522	4.44E-02	9.04E-05	2.91E-05	0.14
Ever breastfed	*AC114730.3*	2q37.3	192	3.33E-04	1.21E-01	6.85E-05	0.17
Ever breastfed	*AKAP3*	12p13.32	695	5.91E-04	2.51E-03	3.58E-05	0.17
Smoking status	*PMS2P3*	7q11.23	217	1.06E-05	2.80E-01	4.00E-05	0.17
Smoking status	*RP11–7I15.4*	11q14.1	350	5.94E-03	5.11E-04	6.94E-05	0.17

^a^FDR correction was conducted using the Benjamini–Hochberg (BH) approach, for each environmental factor.

## Discussion

In this large transcriptome-informed investigation of GxE interactions in breast cancer, we systematically studied the interactions between predicted gene expression and fourteen behavioral and environmental risk factors. No interaction remained statistically significant after adjusting for number of tests. However, we identified seven interactions between genes and environmental factors, including age at first full-term pregnancy, age at menarche, breast feeding history, smoking status, and use of OCs, as suggestive findings with FDR-corrected *P* < 0.20. Our findings did not support a significant role played by gene expression in modifying the associations between established risk factors and breast cancer risk.

The strongest interaction identified was between the *C13orf45* gene and age at the first full-term pregnancy. *C13orf45*, or *LMO7DN*, is a long noncoding RNA (lncRNA) located downstream of the LIM domain only protein 7 (*LMO7*). Few studies have directly focused on the function of *C13orf45* gene. The expression of *LMO7* has been found to play an important role in skeletal muscle transcription and cardiac development (72–74). Irregular expression of the *LMO7* gene has been linked to multiple types of cancer, including breast, thyroid and lung (75–78). Specifically, Hu and colleagues reported that the knockdown of *LMO7* gene in the breast cancer cell line MDA-MB-231 could impair cell migration (76). In the same study, the upregulation of *LMO7* was also found in the stroma of invasive breast carcinoma, which presumably correlated with the expression of serum response factors that regulate muscle and actin cytoskeleton functions. Epidemiologic studies have consistently shown the positive association between later age at first birth and higher incidence of breast cancer (79–81), which can at least be partially explained by pregnancy-induced changes in sex hormones. Earlier differentiation of mammary epithelium induced by estrogen and progestogen at pregnancy can reduce the susceptibility of neoplastic transformation and lower the subsequent disease risk (82). However, there is no direct evidence that this mechanism might interplay with the expression of *C13orf45* or *LMO7*, and therefore, functional follow-up would be needed to explore this potential finding further.

Some of the six additional genes with an FDR-corrected *P*_interaction_ < 0.2 identified in our study have previously been linked to breast cancer development. The translocation and fusion of echinoderm microtubule-associated protein-like 4 (*EML4*) and anaplastic lymphoma kinase (*ALK*) have been implicated in various cancers. For example, the *EML4-ALK1* fusion has been observed in patients with non–small cell lung cancer (83–85), as well as in tumor samples from patients with breast and colorectal cancer (86). *ALK* gene was observed to amplify in most inflammatory breast cancer (IBC; ref. 87), a rare form of disease characterized by an early average age of diagnosis, aggressive histopathologic features, and poor survival (88). There is evidence that IBC cases has a higher prevalence of OC use than other breast cancer cases (89), which suggests that *EML4* may interact with the effect of OC use through inflammatory-related pathways. *AKAP3* is a member of A-kinase anchoring proteins, which has been recognized as a cancer-testis antigen for multiple types of cancer, including ovarian, hepatocellular, and colorectal (90–92). In an investigation of 162 tumor and normal tissues of breast, lack of *AKAP3* expression was observed to be significantly associated with triple-negative breast cancer, breast tumor size, tumor stage, and 5-year disease-free survival (93). The *PMS2P3* gene has been suggested to interact through gene expression with *PMS2* (94), a gene linked to poor survival from breast cancer (95). Noticeably, *PMS2P3* gene belongs to the mismatch repair (MMR) system, which has been observed to have a stronger effect among smokers in affecting colorectal cancer risk, relative to the never smokers (96). Further studies are needed to confirm these suggestive interactions and corresponding biological mechanisms with more direct evidence.

None of the suggestive interaction identified in our study has been observed by previous GxE studies of breast cancer. Otherwise, we were not able to replicate any significant interactions reported by the other studies, including for the genes harboring the variants with significant GxE interaction. This inconsistency could potentially be attributed to various reasons, such as different study populations, analysis approaches and importantly, adjustment for multiple testing. Given the huge number of tests (4,864 genes × 14 environmental risk factors) performed in our analysis, we performed a conservative Bonferroni correction approach and defined a threshold of *P* < 7.34 × 10^−7^ as statistically significant. As this stringent threshold may yield false negative results, we further adopted a more liberal threshold and reported all GxE interactions with FDR-corrected *P* < 0.20 for each environmental factor.

Our study has several strengths. First, to our knowledge this is the first study to incorporate breast tissue specific gene expression models to inform our GxE interaction analysis. Previous research has suggested that breast cancer susceptibility loci are enriched in regulatory regions identified in breast tissue or cell lines (67, 97). Based on this tissue specificity, we utilized genotype and gene expression data from mammary tissue to build gene expression prediction models, and used these models as prior information when assessing GxE interactions. By using a mixed-effects score test which enables the consideration of both fixed and random effects of the interaction, we were able to take into account the effect of genetic variants not involved in gene expression regulation. To avoid potential selection bias, we limited our study population to breast cancer cases and controls from population-based studies. However, our study was based on European ancestry women only, and thus our study conclusions may not be applicable to women with other ancestry. For certain suggestive GxE interactions detected, the results were mainly driven by the random effect component rather than the fixed effect, which made it challenging to explain the mechanisms or pathway underneath. A proportion of the studies included in our analysis adopted the case–control study design, which collected risk factor data based on self-report approaches. Consequently, the risk factor data, although centrally harmonized across all studies, might still be susceptible to recall bias. Our study did not stratify the breast cancer cases by menopausal or estrogen receptor (ER) status and investigate the subtype-specific GxE interaction, which may be a missed opportunity as the disease etiology differs across these subtypes. The results for current use of estrogen-only and estrogen plus progestogen MHT showed evidence of inflated type I error rates, indicating potential issues with distribution or modeling of those risk factors.

In conclusion, our study incorporated information on gene expression to investigate comprehensively the interactions between environmental risk factors and genetic variants on breast cancer risk using a mixed-effects score test approach. Our findings suggest a lack of evidence to demonstrate the role played by gene expression in modifying the associations between established risk factors and breast cancer risk.

## Supplementary Material

Supplementary Figure 1 Quantile-Quantile plot (Q-Q plot) of the aMiSTi p-values for each set of the GxE interactions.

Supplementary Tables 1-3 Supplementary Table 1. Sample Size of each participated study, by case-control status and genotype platform. Supplementary Table 2. Association between fourteen environmental factors and the risk of breast cancer. Supplementary Table 3. Interactions between genes and fourteen environmental factors.

Supplementary Information Funding and acknowledgements.
