# Supplementary material for: A Genome-Wide Gene-Based Gene–Environment Interaction Study of Breast Cancer in More than 90,000 Women
Source: Cancer Res Commun. 2022 Apr 8;2(4):211–9. doi: 10.1158/2767-9764.CRC-21-0119 (PMC9604427; doi:10.1158/2767-9764.CRC-21-0119)

**Supplementary Figure 1. Quantile-Quantile plot (Q-Q plot) of the aMiSTi p-values for each set of the GxE interactions**


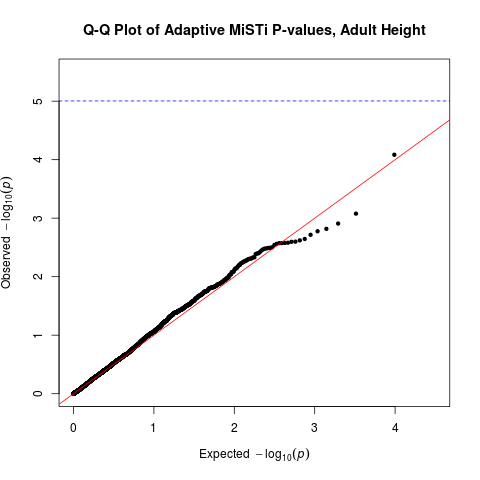


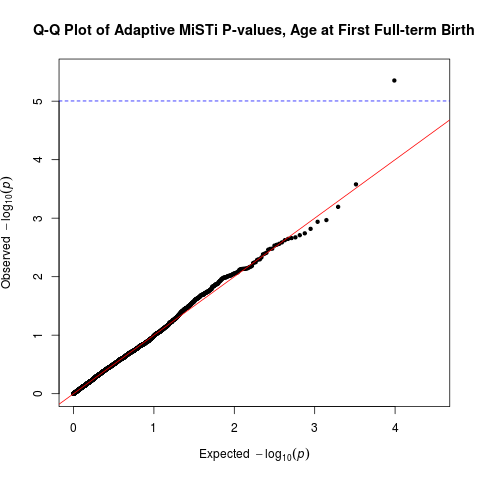

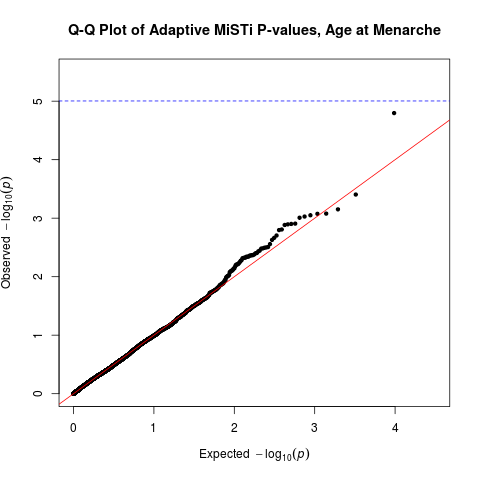

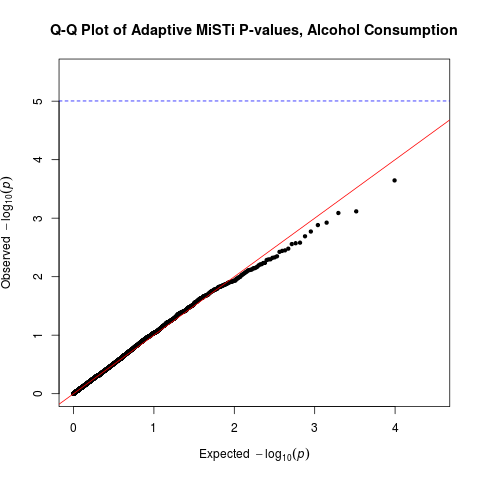

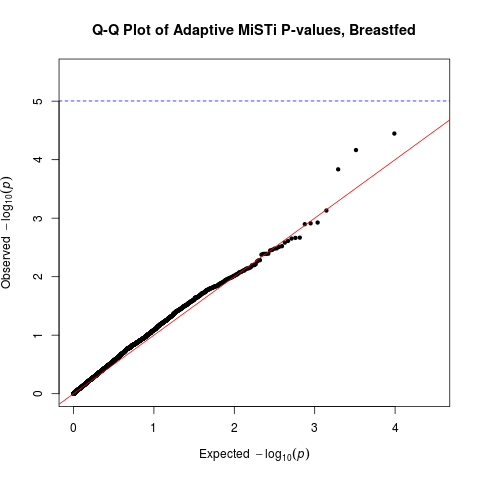

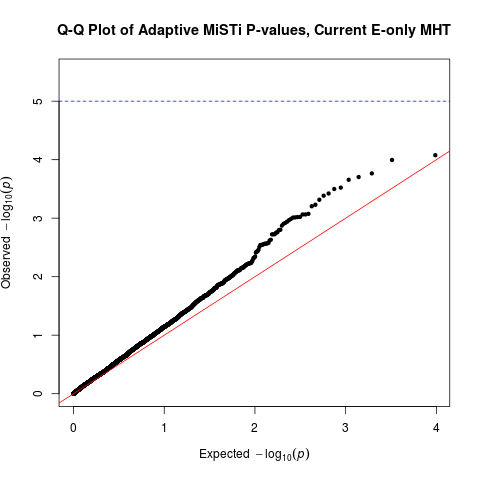


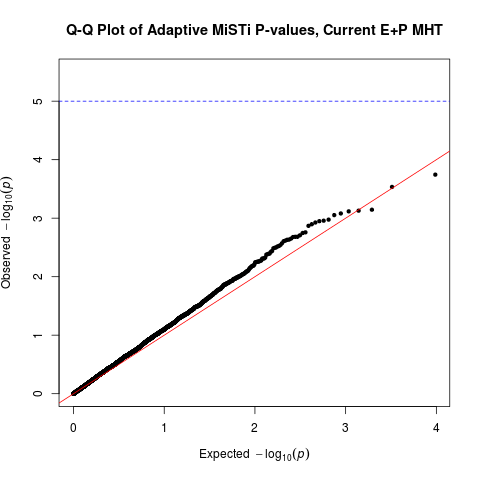


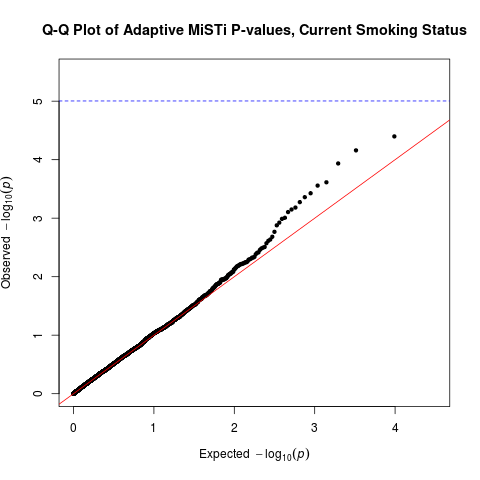

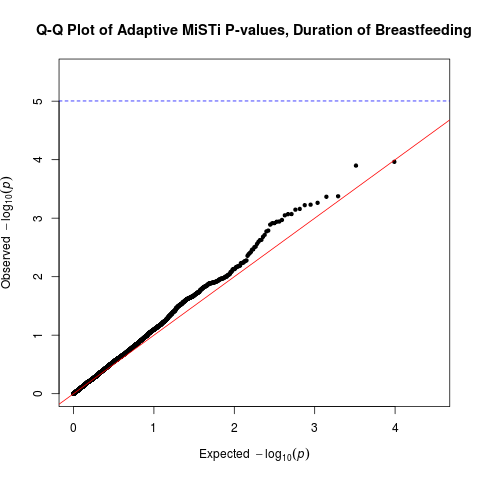

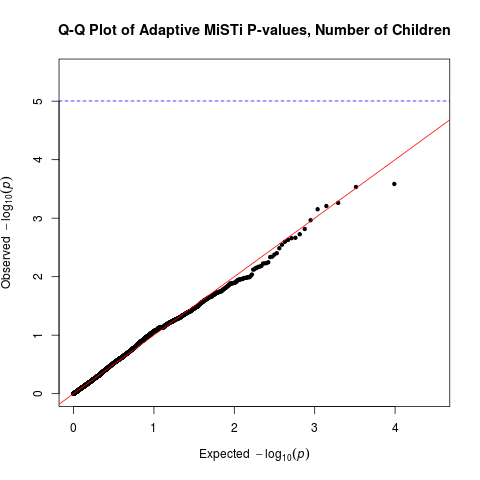

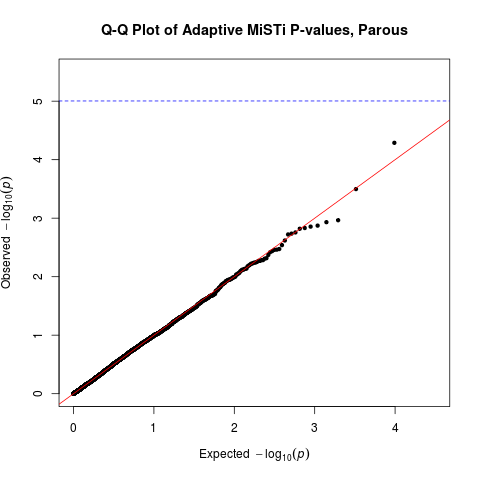

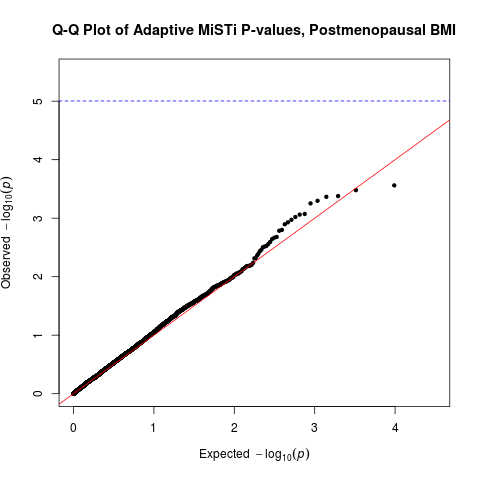

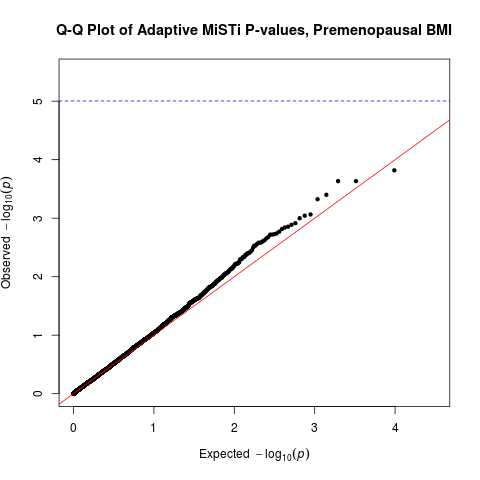

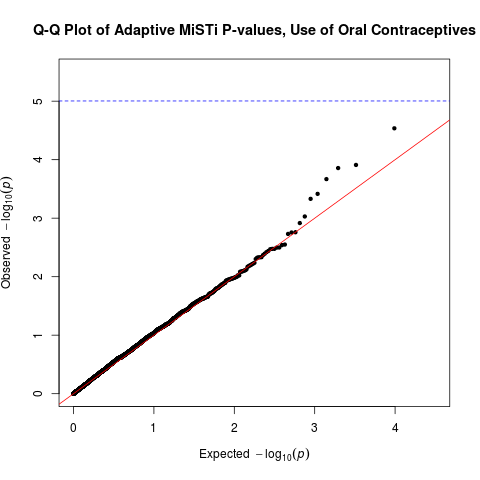

Supplement: Supplementary Figure 1 — Quantile-Quantile plot (Q-Q plot) of the aMiSTi p-values for each set of the GxE interactions. [file crc-21-0119-s01.docx]
